# Sex-specific discrimination of familiar and unfamiliar mates in the Tokay gecko

**DOI:** 10.1007/s10071-024-01896-0

**Published:** 2024-08-07

**Authors:** Marie-Ornélia Verger, Maëlle Devillebichot, Eva Ringler, Birgit Szabo

**Affiliations:** 1 University Sorbonne Paris Nord, Paris, France; 2 Division of Behavioural Ecology, Institute of Ecology and Evolution, University of Bern, Wohlenstrasse 50a, Bern, 3032 Switzerland; 3 Department of Physics, Chemistry and Biology, Linköping University, Linköping, Sweden

**Keywords:** Chemical communication, Mate choice, Recognition, Squamata, Tongue flick, Vomerolfaction

## Abstract

Social animals need to keep track of other individuals in their group to be able to adjust their behaviour accordingly and facilitate group cohesion. This recognition ability varies across species and is influenced by cognitive capacities such as learning and memory. In reptiles, particularly Squamates (lizards, snakes, and worm lizards), chemical communication is pivotal for territoriality, reproduction, and other social interactions. However, the cognitive processes underlying these social interactions remain understudied. In our study, we examined the ability of male and female Tokay geckos (*Gekko gecko*) to chemically differentiate familiar and unfamiliar mating partners. Our findings suggest that both sexes can make this distinction, with males responding more to the odour of a familiar mate, and females responding more to unfamiliar mates. The lizards maintained their discriminatory abilities for two to three weeks but not up to six weeks after separation. This research highlights the efficacy of using odours as social stimuli for investigating social cognition in lizards, a promising avenue to better understand social cognition in these animals.

**Supplementary Information:**

The online version contains supplementary material available at 10.1007/s10071-024-01896-0.

## Introduction

The ability to recognise individuals is most important for social animals in order to adjust their behaviour appropriately in recurring encounters depending on the identity of the conspecific (e.g. kin, group or no a member, familiar/potential new mating partner or competitor; Yorzinski 2017) and the context (e.g. parental care, courtship or territory defence; Edward 2015; Yorzinski 2017). Individuals of highly social species that live in complex group structures might be able to recognize and differentiate single individuals (e.g. Bull et al. 2000; Sayigh et al. 1999), which is expected to facilitate group cohesion (Rios and Kraenkel 2017). In turn, individuals from other, less social species, might only be able to discriminate categories of individuals such as familiar versus unfamiliar (Yorzinski 2017). Variation in the ability to recognise individuals across taxa is caused by variation in the underlying cognitive capacity, such as learning or memory (Yorzinski 2017). These capacities have been largely tested and confirmed in mammals (e.g. Gilfillan et al. 2016; Proops et al. 2009), but information in other taxa is more scarce (Houck 2009).

Depending on the species, the cues used to discriminate between individuals can involve visual signals (e.g. Parr et al. 2000), acoustic signals (e.g. Miller 1979; Nichols and Yorzinski 2016; Warrington et al. 2015), olfactory signals such as pheromones (e.g. Kaur et al. 2014; Keller et al. 2009; Péron et al. 2014), or combinations of different sensory modalities (e.g. Gilfillan et al. 2016; Proops et al. 2009). Across taxa, chemicals are used for social communication (Keller et al. 2009);for example, in the crayfish *Orconectes limosus*, juveniles chemically discriminate their mothers from unfamiliar females, preferentially associate with them and females are less likely to prey on their own offspring (Mathews 2011). Furthermore, zebra finch chicks (*Taeniopygia guttata*) also chemically discriminate their parents from unfamiliar adults and beg more towards chemicals originating from their parents (Caspers et al. 2017). Moreover, male but not female checkerboard worm lizards (*Trogonophis wiegmanni*) discriminate sex based on chemical cues, and both sexes discriminate a familiar from an unfamiliar mating partner (Martin et al. 2020). Research in several mammalian species demonstrated that the main olfactory system is involved in the recognition of mates (Bakker 2003; Keller et al. 2009), and the vomeronasal system is the main pathway for chemical recognition in reptiles (Martín and López 2011; Mason 1992). Therefore, the olfactory system is the prominent pathway involved in chemical communication across taxa.

Reptiles strongly depend on their chemosensory perception, and therefore, have a highly developed vomeronasal system (Martín and López 2011; Mason 1992; Scott et al. 2015). Chemoreception is deeply involved in reptile territoriality, reproduction, recognition of individuals, choice of partner, and social communication (Cooper 1994; Martín and López 2011; Mason 1992). Chemicals might be sequestered by the skin but also by specialised glands such as the femoral and precloacal glands; the latter being more active in males and during the breeding season (Cooper and Burghardt 1990; Houck 2009; Martín and López 2011; Mason 1992; Weldon et al. 2008). To perceive these odours, reptiles, specifically Squamates (lizards, snakes and worm lizards), are known to perform tongue flicks (Cooper 1994). A tongue flick is a protrusion of the tongue out of the mouth to act as a sampling instrument for the collection of volatile and non-volatile compounds (Cooper 1994). Using the tongue flick quantification method, studies have shown that some lizards species can discriminate their own odour from that of conspecifics (e.g. Alberts 1992; Aragón et al. 2001; Cooper 1996; Cooper et al. 1999; Mangiacotti et al. 2020; Moreira et al. 2006; Steele and Cooper 1997; Szabo and Ringler 2023), familiar from unfamiliar same-sex conspecifics (e.g. Aragón et al. 2001; Font and Desfilis 2002), familiar from unfamiliar conspecifics of the opposite sex (e.g. Cooper 1996; Steele and Cooper 1997), kin from non-kin (e.g. Bull et al. 1994, 2000; Main and Bull 1996) and can even discern dominance status (e.g. Moreira et al. 2006). Even though these studies have already demonstrated the capacities of different lizard species to use chemicals for social communication, so far, research is limited specifically lacking investigations in social species that live in groups and perform parental care. Differences in the extent of discrimination ability as well as memory might be adaptive depending on the level of sociality (e.g. short term versus long term groups) or mating system (e.g. monogamy versus polygamy) across species. For example, species that live in short-term groups with seasonal monogamy might only discriminate their mate from unfamiliar individuals of the opposite sex and remember them for only as long as their chemical signature is reinforced (i.e. through habituation). While species that show long-term monogamy and those living in large groups might benefit from individual recognition and long-term memory to improve group cohesion (e.g. Bull 1994; Bull et al. 2000; Rios and Kraenkel 2017; Steele and Cooper 1997).

In this study, we aim to provide insights into the ability of Tokay geckos (*Gekko gecko*), a social lizard species, to discriminate the odour of a familiar from an unfamiliar mate. Male and female Tokay geckos stay together for at least one breeding season (Grossmann 2007) which lasts about four months (Nijman and Shepherd 2015). They form family groups with their offspring for which they provide biparental care both during the egg stage and after hatching (Grossmann 2007). Based on previous studies in lizards (e.g. Cooper 1996; Steele and Cooper 1997) and together with the fact that Tokay geckos form pairs, we expected that geckos could discriminate between familiar and unfamiliar mates using chemical cues. Similar to previous studies, we expected that geckos would show more tongue-flicks towards stimuli from unfamiliar mates, that individuals would produce more tongue flicks toward social stimuli than controls, and that they would show lower responses towards their own odour compared to other social odours (e.g. Cooper 1996; Steele and Cooper 1997; Martin et al. 2020; Szabo and Ringler 2023). Furthermore, we wanted to know for how long individuals are able to discriminate odours. We expected that the geckos’ ability to discriminate would decrease over time: (1) If geckos only discriminate between a familiar and unfamiliar mate for a short period of time (first two weeks; e.g. Glaudas 2004; Herzog et al. 1989), habituation (short-term change that at least partially reverts back to its original state after a certain period of time with no stimulation; Thorpe 1963; Rankin et al. 2009) is the more likely underlying cognitive process. Therefore, we expected to see no difference in the response rate towards the odour of a familiar and unfamiliar mate already in the first session (after 2 weeks of separation), because habituation would decline rapidly when no longer exposed to the odour of a familiar mate. (2) On the other hand, if discrimination lasts for an extended period of time (e.g. a slow decrease with individuals potentially still able to discriminate after four months), the more likely underlying cognitive processes is learning and memory. To this end, we presented lizards with five stimuli: a blank control (untreated filter paper), pungency control (peppermint essential oil), their own odour (to which they are constantly exposed and should respond similarly across time), the odour of a familiar mate, and the odour of an unfamiliar, potential new mate. To assess how discrimination ability changes over time, we presented lizard with the stimuli four times across four months.

## Methods

### Animals, captive conditions and husbandry

14 adult, naïve, captive bred Tokay geckos were tested in this study: 7 males (Snout vent length range across the testing period = 14.62–16.01 cm), and 7 females (Snout vent length range across the testing period = 12.64–13.88 cm). All individuals were purchased from different breeders and were between 3 and 8 years old. Sexes were determined by looking at the presence (for males) or absence (for females) of femoral glands (Grossmann 2007). Lizards were kept in pairs from January 2022 to January 2023, and were separated two weeks prior to the start of the experiment.

Animals were housed singly in terraria with a bioactive set-up: females tanks measured 45 L × 45 B × 70 H cm and males tanks measured 90 L × 45 B × 100 H cm. Terraria were made of rigid foam plates with a net top and glass front doors. They were fitted with a compressed cork wall fixed to the back, cork branches cut in half hooked on the back (functioning as shelters), cork branches allowing lizards to climb, and life plants as enrichment. Each terrarium had a drainage layer of expanded clay, separated by a mosquito mesh from the soil placed on top (organic tropical forest soil; Dragon BIO-Ground). We spread sphagnum moss and autoclaved red oak leaves on the soil as shelter and food for the isopods and earth worms that decompose the faecal material of the lizards. Animals were kept across two rooms. All enclosures were organized on shelves with small enclosures on the top and large enclosures on the bottom. To simulate natural environmental conditions, the room environment was controlled by an automatic system. Animals were exposed to a reversed 12 h:12 h photo period (i.e. light from 6 pm to 6 am, dark from 6 am to 6 pm). The system imitated sunrise and sunset, which were accompanied by changes in temperature reaching approximately 25 °C during night and 31 °C during day. In addition, an UVB light (Exo Terra Reptile UVB 100, 25 W) was provided on top of the terraria during the day. A red light (PHILIPS TL-D 36 W/15 RED) invisible to geckos (Loew 1994) was kept on for 24 h so as to enable experimenters to work with the lizards. Furthermore, lizards could thermoregulate to their optimal body temperature at any time due to a heat mat (TropicShop) attached to the right outer wall of each enclosure, which locally increased the temperature by 4–5 °C. Humidity was kept at 50%, but every 12 h, at 5pm and 4am, 30 s of rainfall (with reverse osmotic water) briefly increased humidity to 100%.

Animals were fed three times per week (Monday, Wednesday, Friday) with between three and five mealworms (*Tenebrio molitor*), cockroaches (*Nauphoeta cinerea*), or adult house crickets (*Acheta domesticus*). In order to provide optimal nutrition to our animals (vitamin D and calcium), the insects were fed with high protein dry cat food (various brands), cricket mix (reptile planet LDT), and fresh carrots. Each gecko was fed with 25 cm long forceps in order to control food intake. Fresh water was supplied ad libitum in a water bowl. Moreover, the geckos were weighed every month and measured (snout vent length) approximately every three months, to track their body condition.

### Set-up

The experiment was conducted from 31st of January to 30th of May 2023. Lizards were tested in a testing tank (45 L x 45 B x 60 H cm; Exo Terra). As our animals are kept in two different rooms, we placed one testing tank in each room. These testing tanks were made of glass (with a mesh top), and covered with a black plastic film on three sides (leaving the front transparent for video recording). Tanks were placed in the middle of the rooms, on a table of 77 cm height, with the transparent front facing a wall at a distance of 100 cm. Two dim white lights (LED, SPYLUX^®^ LEDVANCE 3000 K, 0.3 W, 17 lm) were placed, one on top in the back right corner and one in the middle front, to allow video recording of lizard behaviour in high quality. A GoPro camera (Hero 8; wide mode, 4k resolution, 24 FPS) mounted on a tripod (95 cm height, 55 cm distance from the testing tank) was placed in front of the transparent side. The order in which individuals were tested within a day was randomly assigned, as well as the order of the stimuli they were tested with (but counterbalanced to ensure that the order was different each session but even across individuals each session). Each animal was tested once a day, on non-feeding days (Tuesday and Thursday), for five trials across 2.5 weeks (i.e. session, together 3 Tuesdays and 2 Thursdays), a total of four sessions with an inter-session interval of 19 days.

### Procedure

First, the camera was fixed to the tripod in the room in which the focal individual was housed. Then, the filter paper (either an unused piece for the controls or taken from the enclosure of an individual) was taped to the middle of the back wall of the testing tank (centre of the paper at 21.75 cm from the top and 29.25 cm from the side walls). To make sure the position of the filter paper was always the same, the back wall was marked with a piece of tape. Next, the focal individual was caught in a transparent plastic container (22.8 L x 10.6 B x 7.2 H cm), and placed within the container inside the testing tank (in the middle, directly in front of the back wall). The individual was left alone for 5 min of acclimation after capture. Thereafter, the camera was turned on (activation of the preview), the plastic container’s lid was removed to allow the focal individual to explore the testing tank, and the testing tank’s doors locked. The experimenter then left the room and observed the focal individual live on a smartphone, using the preview of the GoPro quick app (version 11.16). The video recording was started as soon as the focal gecko showed its’ first tongue flick (TF) and lasted for 10 min thereafter (Supplementary video M1). If a lizard did not exit the plastic container within 10 min, the trial was considered as NA and ended (*N* = 7, NA trials were not repeated). At the end of the trial, the individual was caught in the same plastic container and released back into its enclosure. Before the next trial, the testing tank and the plastic container were cleaned using an ample amount of 70% ethanol and whipped dry with paper towels. Everything was left to dry for a minimum of 10 min to allow the alcohol to evaporate. The experimenter washed their hands with water and soap at the end of each trial in order to not contaminate other filter papers with odour remaining from previous trials.

### Stimuli

Each animal was tested with five stimuli: the odour of a familiar mate (kept together and mating for one year but separated 2 weeks before the experiment started), the odour of an unfamiliar mate (potential new mate they had not mated with previously; a different individual was used each session), their own odour (as a social control for which responses should not decrease across time due to constant reinforcement), no odour (C1 - paper control), and peppermint essential oil (farfalla AromaCare) odour (C2 - peppermint control). We included the paper control to make sure the responses of the lizards were consistent over time. We included the peppermint oil control to make sure novelty was not the cause of an increased response rate. All social stimuli were collected using a filter paper (Laboratory filter paper, 12.5 cm diameter, Betzold) pinned to the back wall within enclosures in the sleeping spot 1–5 days before a trial. Due to an error in stimulus collection during repetition three and four, in eight trials the filter paper was left inside an enclosure for only one day while in seven trials it was left for five days (5 males and 4 females; for one male in three trials, for one female in two trials, all other individuals just once; for one day – four times their own odour, three times the familiar mates odour, once the unfamiliar mates odour; for five days – twice their own odour, four times their familiar mates odour and once the unfamiliar mates odour). In all other trials it was left for three days. To collect their own odour, we placed a filter paper in the enclosure of the focal lizard. To collect the odour of the familiar mate, we placed a filter paper in the enclosure of the focal individuals’ familiar mate. Finally, to collect the odour of the unfamiliar mate, we placed a filter paper in the enclosure of a lizard located in the second room with which the focal individual had had no previous contact with. To create the pungency control, we spread peppermint oil onto an unused filter paper using a roll-on in four spots (top, bottom, left and right).

### Data collection

The videos were scored blind as to the presented stimuli (Supplementary video M1). We used the Behavioural Observation Research Interactive Software (BORIS, Version 7.13.9.; Friard and Gamba 2016) to score behaviours performed during trials. We scored (1) TF towards the stimulus (on the filter paper or within an area of one lizard head length around the filter paper), (2) visible TF performed at any other location within the testing tank, and (3) total visible TF. Videos could not be scored blind as to the individual ID. Therefore, approximately 30% of trials were watched by a second observer (80 of 280 trials) to calculate inter-observer reliability. We found very high correlation scores across observers: r_stimulus_ = 0.991 (Pearson correlation, CI_low_ = 0.985, CI_up_ = 0.994, t = 63.654, df = 77, *p* < 0.001), r_other_ = 0. 977 (Pearson correlation, CI_low_ = 0.964, CI_up_ = 0. 985, t = 39.988, df = 77, *p* < 0.001), r_total_ = 0.975 (Pearson correlation, CI_low_ = 0. 961, CI_up_ = 0. 984, t = 38.413, df = 77, *p* < 0.001). As lizard behaviour can be affected by temperature, we also recorded room temperature every 15 min, with an accuracy of 0.1 °C.

### Statistical analyses

Due to a potential asymmetry in information content contained in the odour of males and females (sexually selected femoral gland secretions in males but not in females), we analysed our data separated by sex. We ran one model each for females and males using the number of TF towards the stimulus as the response variable in a censored Bayesian generalised linear mixed model with negative Binomial distribution (GLMM, package *brms*; Bürkner 2017, 2018, 2021). We only analysed TF towards the stimulus as we were not sure if other TF and total TF were impacted by other behaviours such as exploration of novel space (but for completeness, these TF are still included in the raw data file). Trials in which a lizard did not visit the back wall on which the stimulus was presented were coded as censored (= 1; *N* = 34 out of 280 trials) as we assumed that if trials were run longer than 10 min lizards would have eventually visited the filter paper. As fixed effects we included (1) stimulus in interaction with session to understand if there was a stimulus specific change in responses over time, (2) temperature to account for differences in responses due to the ectothermic nature of lizards, and (3) the size of the individual (SVL – snout vent length in cm) to account for differences in behaviour based on individual size. Additionally, we included animal identity as the random effect to account for repeated measures. We made sure that model Rhat was 1, that the ESS was above 2000 and checked the density plots and correlation plots to ensure that the models had sampled appropriately. We used a diffuse normal prior with a mean of 0 and a standard deviation of 1 and ran 4 chains per model of 5000 iterations each with a thinning interval of 1 (default settings). To investigate the results of interactions we used *post hoc* Bayes Factor pairwise comparisons from the package *pairwiseComparisons* (PC, Patil 2019). Finally, we were also interested to see if individuals (both males and females pooled) were consistent in their response to these different stimuli over time while showing distinct differences from each other. We calculated adjusted repeatability of the stimulus directed TF adjusting for stimulus using the package *rptR* (Stoffel et al. 2017). All statistical analyses were run in R version 4.2.2 (R Core Team 2022). To determine the presence of a biologically relevant difference, we used Bayes Factors (BF) and only report results for which BF are above 1 indicating more support for a difference, while numbers below 1 represent a higher likelihood of no difference (Schmalz et al. 2023).

**Fig. 1:**
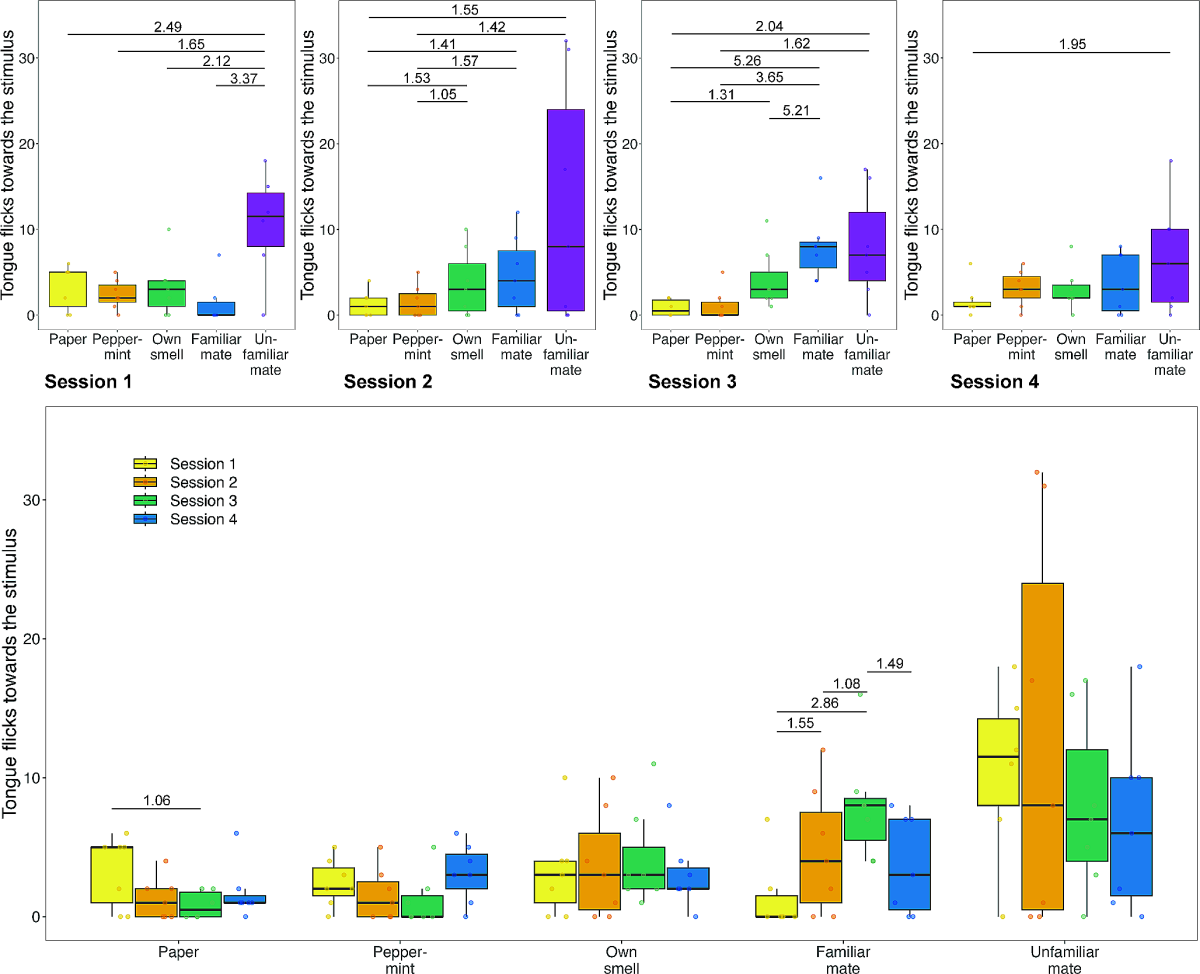
Number of TF females showed towards the different stimuli. Top: Data split into sessions. Bottom: All data split into session per stimulus. The bold line within the boxes shows the median, the upper box edges show the upper quartile, the lower box edges the lower quartile, the top whisker ends show the maximum and the bottom whisker ends the minimum (outliers are not shown). Points represent individual responses. We highlight differences based on the Bayes factor (numbers above lines) and only present those with a Bayes factor above 1. Numbers above 1 indicate more support for a difference, while numbers below 1 represent a higher likelihood of no difference

## Results

**Fig. 2:**
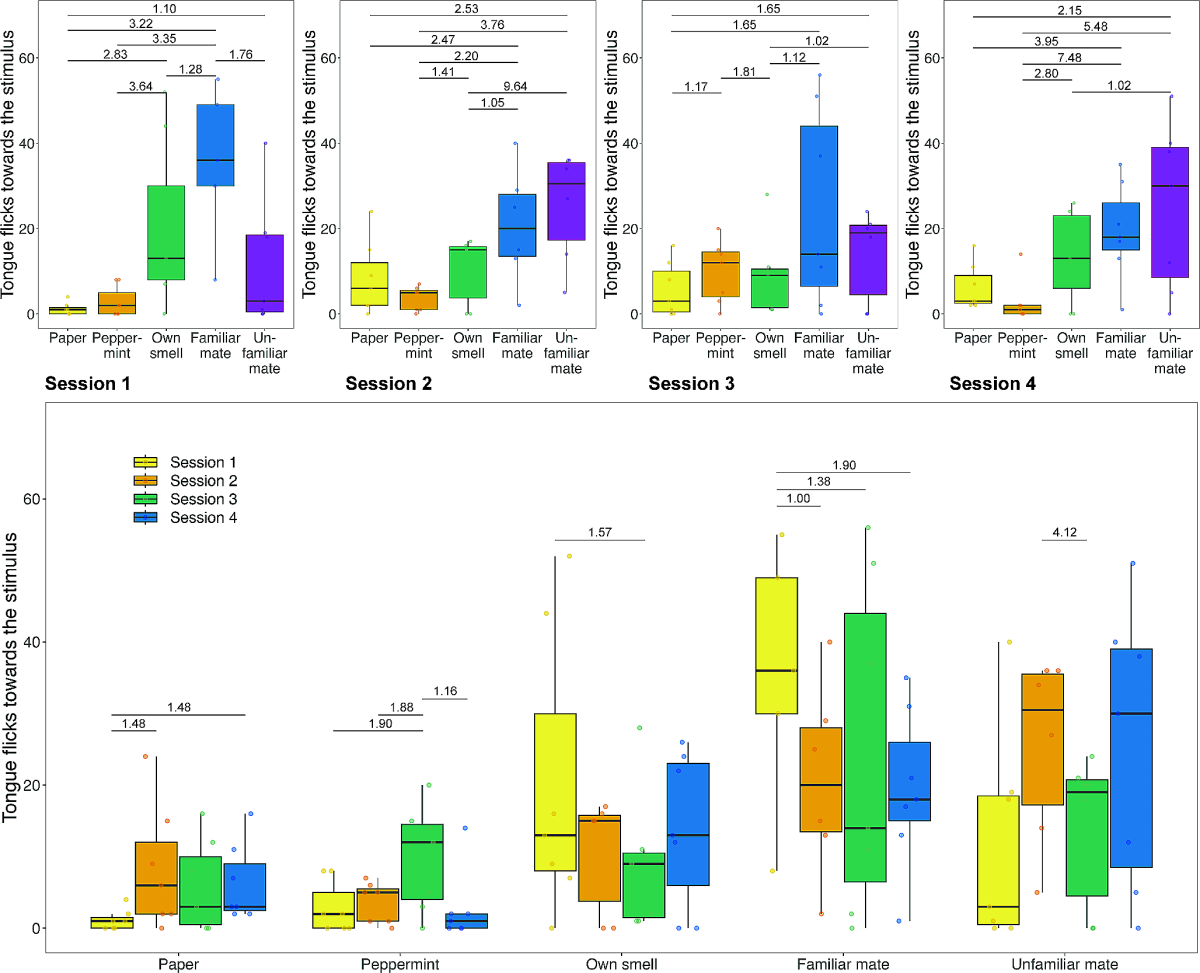
Number of TF males showed towards the different stimuli. Top: Data split into sessions. Bottom: All data split into session per stimulus. The bold line within the boxes shows the median, the upper box edges show the upper quartile, the lower box edges the lower quartile, the top whisker ends show the maximum and the bottom whisker ends the minimum (outliers are not shown). Points represent individual responses. We highlight differences based on the Bayes factor (numbers above lines) and only present those with a Bayes factor above 1. Numbers above 1 indicate more support for a difference, while numbers below 1 represent a higher likelihood of no difference

Overall, females showed less TF than males across all stimuli (mean ± SD; paper: females = 1.81 ± 2.00, males = 5.36 ± 6.30; peppermint: females = 2.07 ± 1.96, males = 4.75 ± 5.60; own odour: females = 3.54 ± 3.24, males = 13.44 ± 13.08; familiar mate: females = 4.59 ± 4.31, males = 30.07 ± 26.93; unfamiliar mate: females = 9.44 ± 8.88, males = 18.92 ± 15.67). We found evidence that both males and females were able to discriminate between the presented social odours; however, in a sex specific manner that changed over time (Figs. 1 and 2; electronic supplementary Table S1). Females produced more TF towards the odour of an unfamiliar mate compared to all other stimuli presented in session 1, but we found no evidence of a difference between any other presented stimuli (Fig. 1; Table 1). In session 2, females produced more TF towards social stimuli than the paper control and the peppermint control, but we found no evidence that they discriminated across social stimuli (Fig. 1; Table 1). In session 3, females produced more TF towards the social stimuli compared to the paper control, and the social stimuli except their own odour compared to the peppermint control as well as the odour of a familiar mate compared to their own odour (Fig. 1; Table 1). Finally, in session 4, females only produced more TF towards the odour of an unfamiliar mate compared to the paper control but we found no difference between all other stimuli (Fig. 1; Table 1). Neither temperature (GLMM, estimate = 0.070, CI_low_ = -0.196, CI_up_ = 0.336, BF = 0.162) nor size (GLMM, estimate = -0.232, CI_low_ = -0.606, CI_up_ = 0.138, BF = 0.495) had an effect on the response rate of females (electronic supplementary Table S1).

For males, we found evidence that, in session 1, they produced more TF towards social odours compared to the paper control, towards their own and the odour of a familiar but not an unfamiliar mate compared to the peppermint control, as well as towards the familiar mate compared to their own odour and the familiar mate compared to the unfamiliar mate (Fig. 2; Table 1). In session 2, males produced more TF towards the odour of a familiar and unfamiliar mate but not their own odour compared to the paper control, towards all social odours compared to the peppermint control as well as towards the odour of a familiar and unfamiliar mate compared to their own odour. In session 3, males produced more TF towards the peppermint control, the odour of a familiar and unfamiliar mate compared to the paper control as well as towards the peppermint control, the familiar and unfamiliar mate compared to their own odour (Fig. 2; Table 1). In session 4, males produced more TF towards the odour of a familiar and unfamiliar mate compared to the paper control, more TF towards all social stimuli compared to the peppermint control and more TF towards the odour of an unfamiliar mate compared to their own odour (Fig. 2; Table 1). Neither temperature (GLMM, estimate = 0.121, CI_low_ = -0.174, CI_up_ = 0.424, BF = 0.205) nor size (GLMM, estimate = -0.455, CI_low_ = -1.493, CI_up_ = 0.672, BF = 0.847) had an effect on the response rate of males (electronic supplementary Table S1).

**Table 1:** Estimates and test statistics comparing the number of tongue flicks directed towards the different stimuli across sessions separated by females and males. We report median (estimate) and credible intervals (HPD interval) from the posterior of the model. Results with a higher support for a difference are highlighted in bold (BF > 1). HPD – higher posterior density, C1 – blank paper control, C2 – peppermint control

FEMALES					
Session 1	Difference	Estimate	Lower HPD Interval	Upper HPD Interval	Bayes Factor
	C1 – C2	0.940	-1.43	3.51	0.64
	C1 – familiar mate	1.28	-1.36	4.06	0.77
	C1 – own odour	0.950	-1.28	3.50	0.64
	**C1 – unfamiliar mate**	**-5.37**	**-10.8**	**0.565**	**2.49**
	C2 – familiar mate	0.255	-2.45	3.15	0.41
	C2 – own odour	-0.016	-2.43	2.56	0.40
	**C2 – unfamiliar mate**	**-6.10**	**-14.5**	**1.87**	**1.65**
	**Familiar mate – unfamiliar mate**	**-7.01**	**-13.3**	**-0.240**	**3.37**
	Own odour – familiar mate	0.241	-2.01	2.48	0.42
	**Own odour – unfamiliar mate**	**-6.44**	**-13.5**	**1.18**	**2.12**
Session 2	C1 – C2	-0.203	-0.808	0.320	0.52
	**C1 – familiar mate**	**-2.71**	**-6.42**	**0.838**	**1.41**
	**C1 – own odour**	**-1.89**	**-4.26**	**0.511**	**1.53**
	**C1 – unfamiliar mate**	**-9.06**	**-20.3**	**1.92**	**1.55**
	**C2 – familiar mate**	**-2.48**	**-5.53**	**0.443**	**1.57**
	**C2 – own odour**	**-1.63**	**-4.17**	**0.755**	**1.05**
	**C2 – unfamiliar mate**	**-8.62**	**-20.3**	**1.90**	**1.42**
	Familiar mate – unfamiliar mate	-5.80	-18.8	6.60	0.66
	Own odour – familiar mate	-0.763	-5.39	3.60	0.38
	Own odour – unfamiliar mate	-6.85	-17.9	3.99	0.99
Session 3	C1 – C2	-0.339	-2.19	1.31	0.43
	**C1 – familiar mate**	**-6.34**	**-11.5**	**-0.708**	**5.26**
	**C1 – own odour**	**-2.68**	**-6.54**	**1.01**	**1.31**
	**C1 – unfamiliar mate**	**-5.79**	**-12.5**	**1.07**	**2.04**
	**C2 – familiar mate**	**-5.77**	**-11.0**	**-0.383**	**3.65**
	C2 – own odour	-2.14	-6.49	1.73	0.82
	**C2 – unfamiliar mate**	**-5.40**	**-12.1**	**1.35**	**1.62**
	Familiar mate – unfamiliar mate	0.011	-6.06	5.69	0.37
	**Own odour – familiar mate**	**-3.27**	**-5.98**	**-0.524**	**5.21**
	Own odour – unfamiliar mate	-2.67	-9.20	3.28	0.65
Session 4	C1 – C2	-1.05	-3.65	1.35	0.57
	C1 – familiar mate	-1.47	-5.21	2.16	0.54
	C1 – own odour	-0.960	-3.52	1.43	0.53
	**C1 – unfamiliar mate**	**-4.12**	**-8.73**	**0.433**	**1.95**
	C2 – familiar mate	-0.404	-3.72	2.80	0.37
	C2 – own odour	0.114	-2.86	3.02	0.35
	C2 – unfamiliar mate	-2.65	-8.39	2.57	0.68
	Familiar mate – unfamiliar mate	-2.20	-7.98	2.87	0.55
	Own odour – familiar mate	-0.478	-3.15	1.96	0.40
	Own odour – unfamiliar mate	-2.78	-8.77	3.04	0.62
**MALES**					
Session 1	C1 – C2	-1.41	-4.33	1.24	0.77
	**C1 – familiar mate**	**-40.7**	**-79.3**	**-2.58**	**3.22**
	**C1 – own odour**	**-18.2**	**-37.3**	**1.34**	**2.83**
	C1 – unfamiliar mate	-9.40	-24.2	4.49	1.10
	**C2 – familiar mate**	**-38.4**	**-74.8**	**-0.317**	**3.35**
	**C2 – own odour**	**-16.9**	**-32.3**	**-0.411**	**3.64**
	C2 – unfamiliar mate	-7.70	-22.0	5.84	0.82
	**Familiar mate – own odour**	**19.5**	**-6.83**	**48.9**	**1.28**
	**Familiar mate – unfamiliar mate**	**28.6**	**-4.57**	**62.7**	**1.76**
	Own odour – unfamiliar mate	6.95	-13.2	28.2	0.53
Session 2	C1 – C2	4.41	-3.16	12.5	0.94
	**C1 – familiar mate**	**-21.1**	**-43.5**	**3.54**	**2.47**
	C1 – own odour	-1.87	-11.2	6.45	0.47
	**C1 – unfamiliar mate**	**-14.3**	**-29.3**	**0.659**	**2.53**
	**C2 – familiar mate**	**-25.6**	**-54.5**	**2.81**	**2.20**
	**C2 – own odour**	**-6.51**	**-16.4**	**2.76**	**1.41**
	**C2 – unfamiliar mate**	**-19.7**	**-37.2**	**0.275**	**3.76**
	**Familiar mate – own odour**	**17.0**	**-10.3**	**47.1**	**1.05**
	Familiar mate – unfamiliar mate	5.34	-19.8	32.7	0.46
	**Own odour – unfamiliar mate**	**-13.4**	**-21.0**	**-3.32**	**9.64**
Session 3	**C1 – C2**	**-2.80**	**-7.09**	**1.36**	**1.17**
	**C1 – familiar mate**	**-10.6**	**-24.1**	**3.74**	**1.65**
	C1 – own odour	-0.192	-3.67	3.28	0.38
	**C1 – unfamiliar mate**	**-6.64**	**-15.1**	**1.24**	**1.69**
	C2 – familiar mate	-7.46	-21.9	5.27	0.85
	**C2 – own odour**	**2.64**	**-0.452**	**5.71**	**1.81**
	C2 – unfamiliar mate	-3.37	-12.2	4.75	0.59
	**Familiar mate – own odour**	**10.1**	**-5.59**	**26.2**	**1.12**
	Familiar mate – unfamiliar mate	3.81	-7.76	17.5	0.49
	**Own odour – unfamiliar mate**	**-5.92**	**-16.3**	**3.59**	**1.02**
Session 4	C1 – C2	2.58	-3.37	8.88	0.59
	**C1 – familiar mate**	**-10.8**	**-20.5**	**-0.550**	**3.95**
	C1 – own odour	-5.61	-16.0	5.11	0.75
	**C1 – unfamiliar mate**	**-15.2**	**-32.0**	**1.26**	**2.15**
	**C2 – familiar mate**	**-14.4**	**-24.5**	**-3.39**	**7.48**
	**C2 – own odour**	**-9.13**	**-18.4**	**-0.243**	**2.80**
	**C2 – unfamiliar mate**	**-19.4**	**-34.6**	**-3.55**	**5.48**
	Familiar mate – own odour	4.30	-3.27	12.2	0.72
	Familiar mate – unfamiliar mate	-3.92	-19.4	10.5	0.44
	**Own odour – unfamiliar mate**	**-8.88**	**-22.1**	**3.79**	**1.02**

We found evidence that responses changed over time. In females, we found evidence that TF towards the paper control decrease from session 1 to session 3 (Fig. 1; Table 2). Furthermore, we found that females increased their TF towards the odour of a familiar mate from session 1 to session 2, session 1 to session 3 and session 2 to session 3 but decreased responses from session 3 to session 4 (Fig. 1; Table 2). In males, we found an increase in the response towards the paper control from session 1 to session 2 and from session 1 to session 4 (Fig. 2; Table 2). Males increased their TF towards the peppermint control from session 1 to session 3, session 2 to session 3 but decreased responses again from session 3 to session 4 (Fig. 2; Table 2). Males also produced less TF towards their own odour from session 1 to session 3 (Fig. 2; Table 2). Males also reduced the number of TF shown towards the odour of a familiar mate from session 1 to session 3 and 4 as well as reduced responses towards the odour of an unfamiliar mate from session 2 to 3 (Fig. 2; Table 2). Finally, we found lizards stimulus directed TF to be repeatable at  *R* = 0.183 (credible interval = 0.062–0.284).

**Table 2:** Estimates and test statistics comparing the relative number of tongue flicks directed towards the different stimuli across sessions separated by females and males. We report median (estimate) and credible intervals (HPD interval) from the posterior of the model. Results with a higher support for a difference are highlighted in bold (BF > 1). HPD – higher posterior density

FEMALES				
C1 – blank paper control				
Difference	Estimate	Lower HPD Interval	Upper HPD Interval	Bayes Factor
Session 1–2	1.33	-1.35	4.31	0.70
**Session 1–3**	**1.59**	**-0.835**	**4.26**	**1.06**
Session 1–4	0.844	-1.30	3.12	0.59
Session 2–3	0.207	-1.36	1.81	0.40
Session 2–4	-0.460	-2.37	1.44	0.45
Session 3–4	-0.717	-2.99	1.54	0.50
C2 – peppermint control				
Difference	Estimate	Lower HPD Interval	Upper HPD Interval	Bayes Factor
Session 1–2	0.625	-0.780	2.17	0.57
Session 1–3	0.922	-0.740	2.80	0.75
Session 1–4	-0.532	-2.87	1.65	0.41
Session 2–3	0.304	-0.510	1.16	0.52
Session 2–4	-1.13	-4.06	1.54	0.57
Session 3–4	-1.50	-4.38	1.06	0.74
Own odour				
Difference	Estimate	Lower HPD Interval	Upper HPD Interval	Bayes Factor
Session 1–2	-0.338	-4.24	3.91	0.36
Session 1–3	-0.617	-4.90	3.36	0.38
Session 1–4	0.234	-3.13	3.56	0.36
Session 2–3	-0.246	-4.59	3.95	0.36
Session 2–4	0.528	-1.58	2.81	0.41
Session 3–4	0.805	-2.61	4.53	0.41
Familiar mate				
Difference	Estimate	Lower HPD Interval	Upper HPD Interval	Bayes Factor
**Session 1–2**	**-1.59**	**-3.58**	**0.418**	**1.55**
**Session 1–3**	**-5.50**	**-10.9**	**0.075**	**2.86**
Session 1–4	-1.95	-6.65	2.34	0.65
**Session 2–3**	**-3.45**	**-9.03**	**1.71**	**1.08**
Session 2–4	-0.520	-5.09	3.86	0.39
**Session 3–4**	**3.15**	**-0.754**	**7.19**	**1.49**
Unfamiliar mate				
Difference	Estimate	Lower HPD Interval	Upper HPD Interval	Bayes Factor
Session 1–2	0.499	-13.5	14.1	0.38
Session 1–3	1.75	-5.45	9.84	0.44
Session 1–4	1.88	-4.14	8.10	0.50
Session 2–3	1.12	-7.33	10.2	0.39
Session 2–4	1.39	-10.5	13.5	0.39
Session 3–4	0.098	-9.50	9.47	0.37
**MALES**				
C1 – blank paper control				
Difference	Estimate	Lower HPD Interval	Upper HPD Interval	Bayes Factor
**Session 1–2**	**-5.62**	**-13.1**	**1.32**	**1.48**
Session 1–3	-3.38	-9.12	2.17	0.89
**Session 1–4**	**-3.98**	**-9.26**	**1.09**	**1.48**
Session 2–3	1.79	-4.99	8.58	0.43
Session 2–4	1.36	-5.18	8.58	0.40
Session 3–4	-0.398	-4.19	3.25	0.36
C2 – peppermint control				
Difference	Estimate	Lower HPD Interval	Upper HPD Interval	Bayes Factor
Session 1–2	-0.515	-4.11	2.81	0.38
**Session 1–3**	**-5.54**	**-12.2**	**0.682**	**1.90**
Session 1–4	0.116	-4.64	4.78	0.35
**Session 2–3**	**-5.10**	**-10.9**	**0.661**	**1.88**
Session 2–4	0.615	-2.67	3.82	0.39
**Session 3–4**	**5.56**	**-2.01**	**13.1**	**1.16**
Own odour				
Difference	Estimate	Lower HPD Interval	Upper HPD Interval	Bayes Factor
Session 1–2	8.08	-9.74	28.8	0.62
**Session 1–3**	**12.8**	**-2.65**	**29.2**	**1.57**
Session 1–4	7.11	-9.25	25.0	0.64
Session 2–3	3.38	-3.69	11.4	0.70
Session 2–4	-0.943	-8.41	6.04	0.39
Session 3–4	-4.36	-15.2	4.90	0.69
Familiar mate				
Difference	Estimate	Lower HPD Interval	Upper HPD Interval	Bayes Factor
Session 1–2	21.2	-12.1	57.9	1.00
**Session 1–3**	**18.0**	**-4.72**	**43.5**	**1.38**
**Session 1–4**	**24.3**	**-3.97**	**53.2**	**1.90**
Session 2–3	-3.59	-24.1	14.1	0.43
Session 2–4	1.05	-12.2	14.9	0.38
Session 3–4	4.73	-12.9	22.8	0.47
Unfamiliar mate				
Difference	Estimate	Lower HPD Interval	Upper HPD Interval	Bayes Factor
Session 1–2	-8.81	-26.2	6.99	0.85
Session 1–3	-0.593	-12.1	10.2	0.40
Session 1–4	-7.99	-32.1	12.2	0.60
**Session 2–3**	**9.41**	**0.538**	**17.2**	**4.12**
Session 2–4	-0.105	-15.8	15.4	0.40
Session 3–4	-7.35	-23.7	6.86	0.81

## Discussion

The results of our study show that both male and female Tokay geckos can discriminate between familiar and unfamiliar mates. However, males directed more TF toward the odour of a familiar compared to an unfamiliar mate, while females directed more TF towards the odour of an unfamiliar mate. Females, overall, responded less than males to all stimuli, even to the controls. While males responded with more TFs towards social stimuli (familiar, unfamiliar and their own odour) compared to the controls (paper only and peppermint oil) across the whole experiment, females only responded with more TFs towards social stimuli starting from the second session but showed no difference again by the last session. In females, we found no evidence for a change in responses across the four sessions, except an increase in responses towards the odour of a familiar mate. In males, we found changes in response rates across time in all stimuli but most notably a decrease in responses towards the odour of a familiar mate. These results suggest a similar temporal change in responses leading to the recognition of a familiar mate vanishing over time in both males and females. This shift occurs approximately four to six weeks after separation. Lastly, we find that lizards TF behaviour was repeatable at *R* = 0.183 showing that studies looking at TF rate should use a within-subject design to account for differences in TF rate across individuals.

We predicted that geckos could use odours to discriminate between familiar and unfamiliar mates and that they would show more TF towards stimuli from unfamiliar mates as has been demonstrated in male and female broadhead skinks (*Plestiodon laticeps*; Cooper 1996), leopard geckos (*Eublepharis macularius*; Steele and Cooper 1997) and checkerboard worm lizards (Martin et al. 2020). Indeed, our analyses show that both male and female Tokay geckos differentiate between familiar and unfamiliar mates (at least in the first session of the experiment), however, only females showed the expected higher response rate towards the odour of an unfamiliar mate. Males showed higher response rates towards the odour of a familiar mate. Generally, differentiating between familiar and unfamiliar mates in both sexes is beneficial, for example, in the context of mate guarding in males and resulting protection from harassment in females (Cooper 1996; Steele and Cooper 1997). Furthermore, it has been proposed that the ability to discriminate between familiar and unfamiliar mates might allow individuals to increase fitness by preferentially courting new mates (Steele and Cooper 1997). Importantly, the reason for the sex difference in response rate towards the odour of a familiar and unfamiliar mate could be due to Tokay geckos’ social system. Mating, egg deposition as well as parental care are performed in the males’ territory (Grossmann 2007). Recognition of the familiar female by the male might be highly beneficial in the context of parental care (O’Connor and Shine 2004), as unrelated females are a threat to the eggs and offspring (Grossmann 2007) and should, therefore, be guarded against. Interestingly, in the checkerboard worm lizard in which males and females also associate in pairs, males direct more TF towards the odour of a familiar compared to an unfamiliar mate while females direct more TF to the odour of an unfamiliar mate (Martin et al. 2020). These findings further support the idea that the social system shapes TF rates in males and females. Importantly, Tokay gecko males also discriminate their own odour from that of the familiar mate, which demonstrates that males do not just label their mates with their own odour so as to make the discrimination between familiar and unfamiliar. Currently, we lack information about Tokay geckos mating system, details about the benefits of parental care and we have no data on mate choice available. It is, therefore, not possible to interpret the difference in responses across the sexes as a preference for familiar/unfamiliar mates, and calls for more research into these topics.

Overall, males produced more TF than females even towards the controls. Similar low responses in females compared to males were found in broadhead skinks (Cooper 1996) and in the thin tree iguana (*Liolaemus tenuis*; Labra and Niemeyer 1999) which might be related to males producing highly salient odours (from sexually selected femoral glands) which females are adapted to recognise quickly. Even though there seems to be a trend for lower TF rates in females across species, in our study, the lower response rate of females might be related to issues with the method of collecting the odour. Males’ enclosures were bigger and, although we placed the filter papers in their sleeping spot, males might have deposited less odour onto the papers compared to females in smaller enclosures. Furthermore, these issues might have led to differences in stimulus quality increasing response variation in both sexes. In the future, it would be better to place filter paper in a small box together with the individual for a set amount of time to control how much odour is deposited.

We also predicted that geckos would show greater responses toward social stimuli, rather than towards the controls, and among social stimuli, less responses towards their own odour, all of which is supported by our results, but most prominently in males. This is likely linked to the information content of the different stimuli, with controls providing the least information, their own odour being very familiar as part of their environment providing no new information (Szabo and Ringler 2023), and the other social stimuli giving the most information about mating partners. The low response rate towards the peppermint oil control also rules out that novelty was a factor influencing response rates but rather social information encoded in the different social chemical stimuli. We also need to point out that, despite similar response rates to the paper and peppermint control, lizards TF more towards the peppermint oil than to a water control in a previous study (Szabo and Ringler 2023) showing that they can perceive the peppermint oil. It is possible that the low TF rate towards the peppermint oil could be due to the reuse in the current study. In future studies, new pungency control stimuli should be used to avoid lower response rates due to familiarisation.

We also found that males discriminate their own odour from the paper control in the first session (= two weeks after separation) but not in session two to four, while females show the same discrimination only starting from session two. The ability to differentiate between the paper control and their own odour points towards no effect of habituation or sensory adaptation to its constant presence. However, in males, the lack of habituation or sensory adaptation seems to change some time after the removal of the female, potentially because the females’ odour vanishes leaving only the males’ odour behind to which it then habituates. The presence of the females’ odour might act as a discriminative stimulus enabling the male to retain detection of its own odour preventing habituation or sensory adaptation. However, this might be an artefact of captivity due to reduced odour diversity (conspecific and heterospecifics) during single housing. Many lizard species are able to differentiate the odour of individuals of their own species from other, even closely related species (e.g. Cooper and Pèrez-Mellado 2002; Cooper and Vitt 1986; 1987). Therefore, under natural conditions males would likely show a permanent discrimination of their own odour, but this should be investigated in the future. Females, on the other hand, show the opposite change, an increase in their ability to discriminate their own odour from the control after the separation from the male. This points towards a reduced ability in females to recognise their own odour during pair housing which, reverts back in single housing until they habituate to the level we found after long term single housing (Szabo and Ringler 2023). Females are adapted to perceive male femoral gland secretions. During pair formation and parental care, females might not need to discriminate their own odour which only becomes relevant again when they separate from the male to occupy their own territory/ home range. However, this pattern could also be an artefact of low odour diversity in captivity. Therefore, studies on wild individuals are necessary to better understand Tokay geckos ability to detect their own odour during and outside the breeding season.

Our last prediction was that odour discrimination would decrease over time. We predicted a fast decrease if habituation was the main cognitive process, while we predicted a slow decrease if learning and memory were involved. Our results show a similar temporal change in both sexes. Across the four months of the experiment, males TF rate towards the familiar mate decreased to the level of responses towards the unfamiliar mate, while females responses towards the odour of a familiar mate increased to the level of the unfamiliar mate. In both cases, we find that the familiar mate becomes unfamiliar about four to six weeks after separation. Previously, blackbelly garter snakes (*Thamnophis melanogaster*) and cottonmouths (*Agkistrodon piscivorus*) habituated to a threatening stimulus over five days, but showed a recovery of anti-predator behaviour after an average of 14 days with no stimulation (Glaudas 2004; Herzog et al. 1989). Currently, our data do not allow us to differentiate if habituation/dishabituation or learning and memory are responsible for Tokay geckos’ ability to discriminate between familiar and unfamiliar mates. Further studies should be conducted with a shorter time interval to pinpoint the exact moment when the odour of a familiar mate becomes unfamiliar again. Furthermore, males, not just females, should be put into new enclosures to better understand how the deterioration rate of odours affects discrimination ability over time and how exposure to an odour influences this ability after the removal of the source individual. Importantly, our results make ecological sense if we assume that Tokay geckos have a polygamous mating system, in which it is beneficial to mate with multiple mating partners. Depending on the exact point in the breeding cycle, both the ability to discriminate (to guard a mate and perform biparental care) or not (after offspring disperse and before the new breeding season) might be beneficial. However, further studies are needed, especially in the wild, to better understand Tokay gecko mating strategies and the associated cognitive abilities.

In summary, we found that Tokay geckos can discriminate familiar from unfamiliar mates. We find a sex-specific response in which males direct more TF towards the odour of a familiar while females direct more TF towards the odour of an unfamiliar mate. Simultaneously, we find a similar change in responses over time depending on this sex-specific baseline leading to the disappearance of the discrimination four to six weeks after separation. Further research should be done to determine the exact time when a familiar mate becomes unfamiliar again and to understand if this discrimination ability is individual specific or categorial. Additionally, studies in the wild are needed to be able to fully interpret the results of our study. Our method is suitable to study chemical recognition in Tokay geckos, but some aspects of the methodology still need to be improved for future investigations into, for example, kin recognition. Similar to previous work (Szabo and Ringler 2023), we showed that using odours as social stimuli is a good tool to investigate social cognition in this species. Overall, our results provide further evidence that lizards are suitable models to investigate chemical communication, especially in a social context.

## Electronic supplementary material

Below is the link to the electronic supplementary material.

Supplementary Material 1

Supplementary Material 2

## Data Availability

Data availability statement: Data generated during this study are available for download from the Open Science Framework (OSF, https://doi.org/10.17605/OSF.IO/7ECXQ). Code availability statement: All code generated to analyse the collected data is available for download from the Open Science Framework (OSF, https://doi.org/10.17605/OSF.IO/7ECXQ).
