# Supplementary material for: Sex-specific discrimination of familiar and unfamiliar mates in the Tokay gecko
Source: Anim Cogn. 2024 Aug 7;27(1):55. doi: 10.1007/s10071-024-01896-0 (PMC11306719; doi:10.1007/s10071-024-01896-0)
Supplement: Supplementary file 2 — Supplementary Material 2 [file 10071_2024_1896_MOESM2_ESM.docx]

**Supplementary material**

To

“Sex specific discrimination of familiar and unfamiliar mates in the Tokay gecko”

By

Marie Ornelia Verger^a,b^, Maëlle Devillebichot^c^, Eva Ringler^b^, Birgit Szabo^b^

^a^ University Sorbonne Paris Nord, Paris, France

^b^ Division of Behavioural Ecology, Institute of Ecology and Evolution, University of Bern, Bern, Switzerland

^c^ Department of Physics, Chemistry and Biology, Linköping University, Linköping, Sweden

*Correspond to: Birgit Szabo, Division of Behavioural Ecology, University of Bern, Wohlenstrasse 50a, 3032 Bern, Switzerland; email: birgit.szabo@gmx.at,

**Supplementary tables**

**Table S1.** Estimates and test statistics from the censored Bayesian generalised linear model looking at stimulus, session and temperature effects on the number of tongue flicks separated by females and males.

| FEMALES | | | | |
| --- | --- | --- | --- | --- |
| Parameter | Estimate | Estimated error | 95% lower Credible interval | 95% upper Credible interval |
| Intercept | 2.248 | 4.511 | -6.718 | 11.081 |
| C2 – peppermint control | -0.087 | 0.361 | -0.790 | 0.643 |
| Own odour | 0.401 | 0.353 | -0.297 | 1.095 |
| Familiar mate | 0.065 | 0.397 | -0.693 | 0.871 |
| Unfamiliar mate | 1.438 | 0.346 | -0.760 | 2.112 |
| Session 2 | -0.171 | 0.372 | -0.904 | 0.564 |
| Session 3 | -0.382 | 0.382 | -1.131 | 0.361 |
| Session 4 | -0.380 | 0.368 | -1.100 | 0.342 |
| Temperature | 0.070 | 0.136 | -0.196 | 0.336 |
| SVL | -0.232 | 0.187 | -0.606 | 0.138 |
| Interactions | | | | |
| C2 – session 2 | -0.158 | 0.539 | -1.203 | 0.871 |
| Own odour – session 2 | 0.299 | 0.518 | -0.715 | 1.306 |
| Familiar mate – session 2 | 0.541 | 0.530 | -0.501 | 1.577 |
| Unfamiliar mate – session 2 | 0.249 | 0.495 | -0.725 | 1.229 |
| C2 – session 3 | 0.158 | 0.569 | -0.948 | 1.282 |
| Own odour – session 3 | 0.320 | 0.514 | -0.695 | 1.316 |
| Familiar mate – session 3 | 1.264 | 0.534 | 0.205 | 2.291 |
| Unfamiliar mate – session 3 | 0.056 | 0.513 | -0.931 | 1.054 |
| C2 – session 4 | 0.465 | 0.512 | -0.534 | 1.461 |
| Own odour – session 4 | -0.055 | 0.508 | -1.053 | 0.929 |
| Familiar mate – session 4 | 0.629 | 0.528 | -0.406 | 1.664 |
| Unfamiliar mate – session 4 | -0.066 | 0.499 | -1.036 | 0.917 |
| MALES | | | | |
| Parameter | Estimate | Estimated error | 95% lower Credible interval | 95% upper Credible interval |
| Intercept | 5.312 | 9.028 | -13.522 | 22.449 |
| C2 – peppermint control | -0.186 | 0.386 | -0.923 | 0.582 |
| Own odour | 1.213 | 0.340 | 0.549 | 1.872 |
| Familiar mate | 1.827 | 0.344 | 1.156 | 2.493 |
| Unfamiliar mate | 0.830 | 0.363 | 0.128 | 1.554 |
| Session 2 | 0.043 | 0.349 | -0.647 | 0.727 |
| Session 3 | -0.215 | 0.340 | -0.869 | 0.443 |
| Session 4 | -0.187 | 0.368 | -0.917 | 0.530 |
| Temperature | 0.121 | 0.152 | -0.174 | 0.424 |
| SVL | -0.455 | 0.543 | -1.493 | 0.672 |
| Interactions | | | | |
| C2 – session 2 | -0.465 | 0.509 | -1.469 | 0.538 |
| Own odour – session 2 | -0.422 | 0.513 | -1.404 | 0.594 |
| Familiar mate – session 2 | -0.407 | 0.459 | -1.309 | 0.510 |
| Unfamiliar mate – session 2 | 0.313 | 0.481 | -0.623 | 1.274 |
| C2 – session 3 | 0.831 | 0.495 | -0.151 | 1.796 |
| Own odour – session 3 | -0.680 | 0.474 | -1.593 | 0.261 |
| Familiar mate – session 3 | -0.251 | 0.470 | -1.153 | 0.693 |
| Unfamiliar mate – session 3 | 0.340 | 0.515 | -0.659 | 1.369 |
| C2 – session 4 | -0.289 | 0.535 | -1.342 | 0.766 |
| Own odour – session 4 | -0.178 | 0.478 | -1.103 | 0.768 |
| Familiar mate – session 4 | -0.562 | 0.462 | -1.443 | 0.352 |
| Unfamiliar mate – session 4 | 0.553 | 0.483 | -0.383 | 1.499 |
